# Isolation and Purification of a Peptide from *Bullacta exarata* and Its Impaction of Apoptosis on Prostate Cancer Cell

**DOI:** 10.3390/md11010266

**Published:** 2013-01-23

**Authors:** Jianyin Ma, Fangfang Huang, Huanle Lin, Xian Wang

**Affiliations:** School of Food Science and Pharmacy, Zhejiang Provincial Key Engineering Technology Research Center of Marine Biomedical Products, Zhejiang Ocean University, Zhoushan 316000, China; E-Mails: gracegang1985@hotmail.com (F.H.); linhuanle2012@gmail.com (H.L.); wangxin296@gmail.com (X.W.)

**Keywords:** *Bullacta exarata* peptides, isolation and purification, prostate cancer, apoptosis

## Abstract

*Bullacta exarata* was hydrolyzed with trypsin to prepare peptides; Hydrolysates were isolated by ultrafiltration and purified using G-25 gel filtration. The purity of the *Bullacta exarata* was demonstrated by HPLC and its peptide sequence analysis was detected. The effects of BEPT II and BEPT II-1 on the proliferation of PC-3 cells were examined using a MTT assay. BEPT II and BEPT II-1 significantly inhibited the proliferation of PC-3 cells in a time- and dose-dependent manner. Annexin V/PI double staining studies showed exposing PC-3 cells to 5, or 15 mg/mL BEPT II-1 for 24 h increased the percentage of the early stage of apoptotic cells from 11.22% to 22.09%. In addition, typical morphologic changes were observed in the cells with acridine orange/ethidium bromide staining. These data support that BEPT II-1 has anticancer properties and merits further investigation to understand the mechanisms of BEPT II-1-induced apoptosis in PC-3 cells.

## 1. Introduction

Prostate carcinoma (PCa) is one of the most prevalent cancers in men. The rate of PCa-related death increases every year. Prostate carcinogenesis is a multistep process involving progression from localized, low-grade lesions to large, high-grade and metastatic carcinomas. Treatment for prostate cancer may include active surveillance (monitoring for tumor progress or symptoms), surgery, radiation therapy, high-intensity focused ultrasound, chemotherapy, cryosurgery, hormonal therapy, or various combinations of these [1,2,3]. However, chemotherapy and radiation therapy are largely ineffective against advanced prostate cancer [4,5]. In addition, a growing number of studies have shown that some of the cationic antimicrobial peptides (AMPs), which are toxic to bacteria but not to normal mammalian cells, exhibit a broad spectrum of cytotoxic activity against cancer cells [6]. Therefore, developing new therapeutic strategies targeting apoptosis induction would be of real value in controlling the proliferation as well as the invasiveness of advanced PCa. 

*Bullacta exarata*, a kind of shell mollusk, lives on inshore tidal land and is a cooking material popular in coastal region in China. It is composed of a shell and a soft body. It is abundant in coastlines of the China Seas from Hainan to Yellow Sea in eastern China. In our previous study, three isolated peptides from *Bullacta exarata*, BEP-1, BEP-2 and BEP-3 [7], showed activity against *Escherichia coli*, *Staphylococcus aureus* and *Bacillus subtilis*. BEP-1 also showed activity against human pathogen strains (*Staphylococcus epidermidis*, *E. coli* and Methecillin-Resistant *S. aureus*). However, to date there are no studies addressing whether *Bullacta exarata* affects cancer cells.

In the present study, BEPT II-1 was isolated from *Bullacta exarata*. The result showed that BEPT II-1 inhibited PC-3 cell growth and the mechanisms were associated with induction of apoptosis.

## 2. Results and Discussion

### 2.1. Isolation and Purification of Peptide

**Figure 1:**
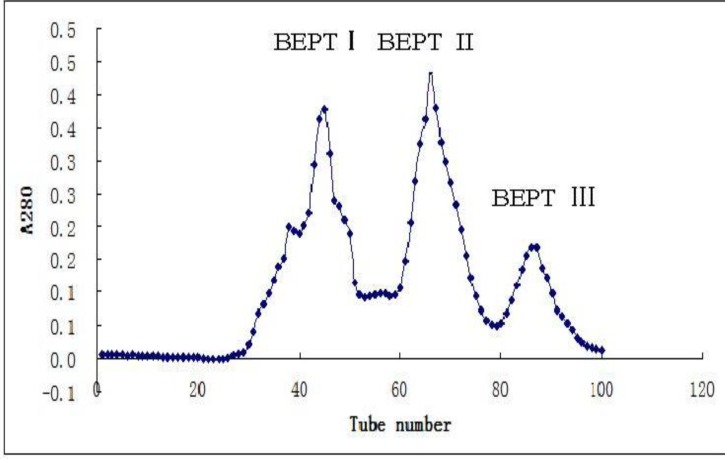
Sephadex G-25 Gel chromatography of the antimicrobial peptide from *Bullacta exarata.*

**Figure 2:**
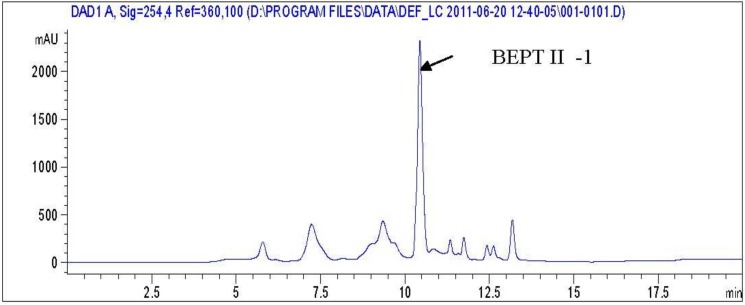
The high performance liquid chromatography (HPLC) chromatogram of BEPTII.

After ultrafiltration using a PM-10 membrane with a molecular weight cut off of 3 kDa, the filtrates of trypsin digestion were loaded on a gel filtration column (Sephadex G-25). After gel filtration, the sample hydrolyzed by trypsin was divided into three peaks which are named BEPT I, BEPT II and BEPT III, respectively (Figure 1). Peak II showed the highest anticancer activity and was further separated by reverse-phase HPLC. As shown in Figure 2, peak A was collected and named BEPT II-1 and its peptide sequence is RAALAVVLGRGGPR and RDGDSCRGGGPV.

### 2.2. BEPT II and BEPT II-1 Inhibits Cell Proliferation of PC-3 Cell Lines

**Figure 3:**
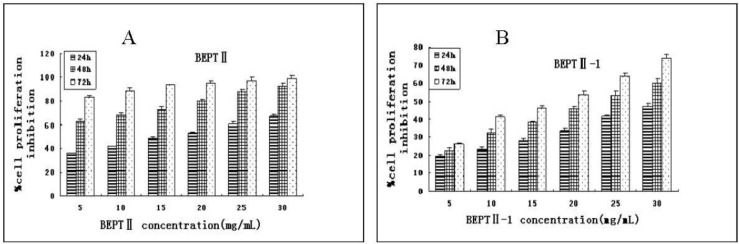
Inhibited proliferation of BEPTII and BEPTII-1-treated PC-3 cells. (**A**) PC-3 cells were treated with 5, 10, 15, 20, 25 and 30 mg/mL BEPTII. (**B**) PC-3 cells were treated with 5, 10, 15, 20, 25 and 30 mg/mL BEPTII-1. Cell proliferation was measured using MTT at 24, 48, and 72 h after BEPTII and BEPTII-1treatment. Results are expressed as mean±SD. Each experiment was performed in triplicate (*n*=3).

The inhibitory effect of BEPT II and BEPT II-1 on the proliferation of PC-3 cells was studied by MTT assay. As shown in Figure 3A, BEPT II inhibited cell proliferation in a dose- and time-dependent manner, and IC_50_ values in PC-3 cells ranged from 14.06 to 1.16 mg/mL after 24 h, 48 h and 72 h of treatment (Table 1). Compared with BEPT II, PC-3 cells were less sensitive to BEPT II-1 at the same concentrations and time and IC_50_ values ranged from 43.97 to14.62 mg/mL (Table 1). We speculated that BEPT II-1 is one of the ingredients of BEPT II; other ingredients in BEPT II might also have anti-proliferation activity on PC-3 cells.

Additionally, the time course of studies of PC-3 cells found that exposure to BEPT II and BEPT II-1 (5–30 mg/mL) for 24 h showed significant inhibitory effects on cell growth and at the same doses, PC-3 cell proliferation decreased markedly at 48 h and 72 h after BEPT II and BEPT II-1 treatment. This result indicates a possibility that BEPT II and BEPT II-1 did not have an immediate toxic effect on PC-3 cells and might induce a series of cellular events which lead to inhibit cell growth and induce cell death. 

**Table 1:** IC_50_ for BEPT II and BEPT II-1.

	IC_50_ (mg/mL)		
	24 h	48 h	72 h
BEPT II	14.06 ± 0.41	3.68 ± 0.12	1.16 ± 0.11
BEPT II-1	43.97 ± 0.34	22.19 ± 0.22	14.62 ± 0.23

### 2.3. Morphologic Observation by Acridine Orange and Ethidium Bromide (AO/EB) Staining

**Figure 4:**
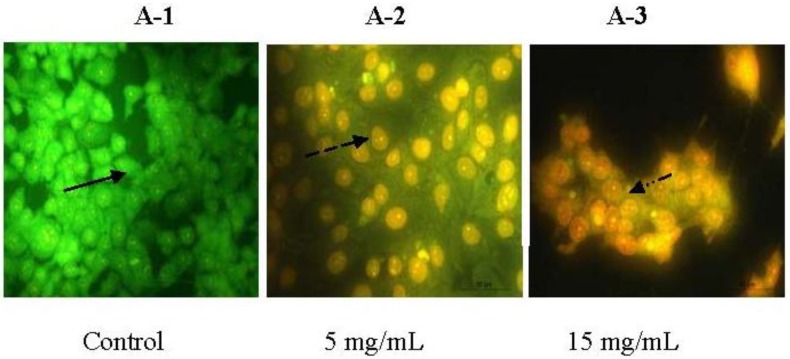
Morphologic observation with acridine orange/ethidium bromide (AO/EB) staining. PC-3 cells (**A**) were treated without (A-1) and with BEPT II-1 at 5 mg/mL (A-2), 15 mg/mL (A-3). (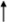) indicates viable cells; (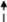) indicates early apoptotic cells; (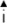) indicates late apoptotic cells. Each experiment was performed in triplicate (*n* = 3) and generated similar morphologic features. Original magnification 400×.

The morphologic changes of the PC-3 cells treated with 5 mg/mL and 15 mg/mL of BEPT II-1 for 24 h were observed by AO/EB staining. BEPT II-1-treated PC-3 showed significant morphologic apoptotic changes. The characterization of morphological changes consisted of reduction in cell volume, cell shrinkage, reduction in chromatin condensation and formation of cytoplasmic blebs [8]. Cells stained green represent viable cells, whereas yellow staining represents early apoptotic cells, and reddish or orange staining represents late apoptotic cells. PC-3 cells treated with 5 mg/mL of BEPT II-1 showed changes in cellular morphology, including chromatin condensation, membrane blebbing, and fragmented nuclei (Figure 4). AO/EB staining revealed that the morphologic features of apoptotic PC-3 cells were dose-dependent.

### 2.4. BEPT II-1 Induces Apoptosis in PC-3 Cells Based on Flow Cytometry Analysis

**Figure 5:**
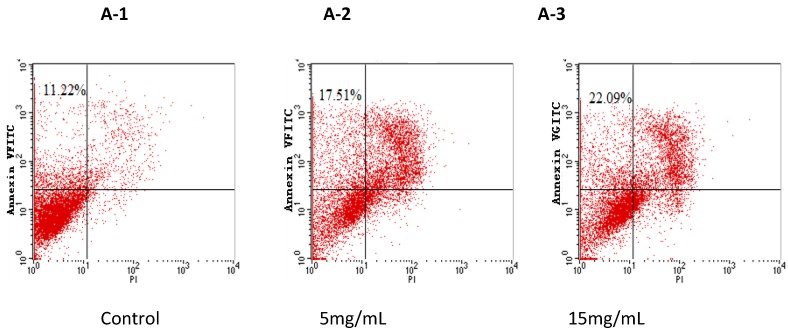
Flow cytometry analysis of PC-3 cells by double-labeling with Annexin-V fluorescein isothiocyanate (FITC) and PI. Quadrants: Lower left—the live cells. Upper left—the early apoptotic cells. Lower right—the necrotic cells, and upper right—late apoptotic cells. Percentages of early apoptotic cells were: (**A-1**) control cells: 11.22% ± 1.2%; (**A-2**) BEPT II-1 (5 mg/mL): 17.51% ± 1.0%; (**A-3**) BEPT II-1 (15 mg/mL): 22.09% ± 2.1%;One representative fluorescence activated cell sorting (FACS) assay of three independent experiments is presented.

Annexin V/PI double staining was performed to characterize whether cell death resulting from BEPT II-1 treatment was caused by apoptosis induction. The upper left quadrants represent the early apoptotic cells (Annexin V binding and propidium iodide [PI] negative). As shown in Figure 5, it was found that the cells treated with BEPT II-1 for 24 h showed increased proportions of Annexin V positive and [PI] negative cells from 11.22% to 22.09%.

Apoptosis is an important homeostatic mechanism that balances cell division and cell death and maintains the appropriate number of cells in the body [9]. Many therapeutic peptides from marine animals, such as dolastatins-10 and 15, promote apoptosis in cancer cells. Moreover, some anticancer drugs cause the death of sensitive cells through the induction of apoptosis. It may mean that there is a cytoxic effect on the PC-3 cells because the proliferation inhibition was over 50% whereas the apoptosis induction was only 20%. The high doses of BEPT II-1 used in this paper really seemed a conundrum to us. We need to further purify BEPT II-1 in order to get a single ingredient to improve the IC_50_.

Two pathways are involved in cell apoptosis, extrinsic and intrinsic pathways [10]. Effector caspases are common to both the extrinsic and intrinsic pathways. Death receptors, through adaptor molecules, recruit initiator caspase-2, -8, or -10, while intrinsic death signals result in the activation of caspase-9 [11]. Initiator caspases cleave procaspases, and are thus able to activate effector caspases (caspase-3, -6 and -7) or to amplify the caspase cascade by increased activation of initiator caspases [12].

Further investigations focused on the mechanisms of BEPT II-1-induced apoptosis in PC-3 cells. 

## 3. Experimental Section

### 3.1. Materials

*Bullacta Exarata* was obtained from Ningbo coastal. Human prostate cancer PC-3 was purchased from Cell bank of Chinese Academy of Science, Shanghai, China. Ultrafiltrate was applied to a column saturated in Sephadex G-25 resin (Amersham Pharmacia biotech, Shanghai, China). The *Bullacta Exarata* peptide fraction was further purified using reverse-phase HPLC on a Primesphere C_18_ column (Phenomenex Co., Ltd., Shanghai, China). The anti-proliferation tumor PC-3 were detected by MTT (Hangzhou Haotian Biotechnology Co., Ltd., Hangzhou, China). Other reagents are analytical reagents made in China.

### 3.2. Enzymatic Hydrolysis

The conditions of the enzymatic hydrolysis were: temperature of 45 °C, pH 8.7, solid-liquid ratio of 1:1 and time of 8 h. Reactions were terminated by heating the solution to 98 °C for 15 min to inactivate the enzyme. The resulting slurry was centrifuged at 10,000 r/min for 20 min.

### 3.3. Isolation and Purification of Anticancer Peptide

#### 3.3.1. Ultrafiltration

*Bullacta* hydrolysates were separated into a large molecular weight fraction and a low molecular weight fraction by ultrafiltration at 4 °C by PM-10 membrane and kept for use in gel filtration. Prior to use, the membrane was activated by spinning 10 mL of distilled water, and the remaining liquid was carefully removed.

#### 3.3.2. Sephadex G-25 Gel Chromatography

Ultrafiltrate was again filtered through a Millipore membrane filter (0.45 μm) and applied to a column (2.6 cm × 100 cm) saturated in Sephadex G-25 resin. Sephadex G-25 column was eluted with distilled water and fractions were collected at 2 min interval with a fraction collector. The absorbance was measured at 280 nm. 

#### 3.3.3. High Performance Liquid Chromatography (HPLC)

The fraction exhibiting the highest anticancer activity was further purified using reverse-phase HPLC on a Primesphere C_18_ column (10 mm × 250 mm) with a linear gradient of acetonitrile (0%–50% for 20 min) containing 0.1% trifluoroacetic acid (TFA) at a flow rate of 1 mL/min. The absorbance of eluent was monitored at 280 nm. 

### 3.4. PC-3 Cells Culture

The hormone-independent PC-3 cell lines were obtained from the China cell bank of the Institute of Biochemistry and Cell Biology. PC-3 cells were cultured in F-12 medium, supplemented with 10% Fetal Calf Serum (FCS) (1% penicillin/streptomycin; 10,000 U/mL penicillin G sodium, 10,000 mg/mL streptomycin sulfate). All cell lines were cultured at 37 °C in a humidified atmosphere in a 5% CO_2_ incubator.

### 3.5. Anti-Proliferative Activity Using MTT Assay

Cell proliferation after treatment with BEPT II-1 and BEPT II was measured by MTT assay. PC-3 cells were seed at a density of 1 × 10^4^ cells/100 μL/well in 96-well Plates and allowed an overnight period for attachment. The medium was then removed and 200 μL of medium FCS was added, followed by BEPT II-1 and BEPT II at a final concentration of 5, 10, 15, 20, 25 and 30 mg/mL. Cells were grown under these conditions for 24, 48, and 72 h at 37 °C in a humidified atmosphere, at 5% CO_2_ incubator. After designated time, MTT was added to each well 4 h before the end of the incubation period, after which conversion of yellow tetrazolium salt to blue thiazol crystals by metabolically active cells was stopped by adding DMSO to each well. The intensity of blue emission in each well was measured using at 450 nm using a microplate reader. All experiments were performed in triplicate and repeated three times.

The percent inhibition of cell proliferation was calculated as follows:

% inhibition = (OD_control_ − OD_treated_)/(OD_control_ − OD_blank_) × 100% (1)

### 3.6. Morphologic Study with Fluorescence Microscope

The AO/EB staining (Acridine orange/Ethidium bromide) method was used to observe the apoptotic morphologic changes. PC-3 cells were suspended at a final concentration of 1 × 10^5^cells/well and cultured above 35 mm slip in 6-well flat-bottomed Plates and allowed to adhere to the bottom of the wells for 24 h before oligopeptide treatment. Cells were exposed to 5, and 15 mg/mL doses of BEPT II-1 for 24 h. F12 medium (10% FCS) was used as a control for the PC-3 cell lines. After designed time, AO/EB mixture [25 μL, containing 100 μg/mL AO and 100 μg/mL EB in PBS (pH 7.4)] was added to cells treated with BEPT II-1. Then the cells were observed under a fluorescence microscope.

### 3.7. Cell Apoptosis Analysis

Cell apoptosis detection was performed by fluorescence activated cell sorting (FACS) analysis using a flow cytometer. The exposure of PS on the extracellular side of the cell membrane was quantified by annexin V-FITC /PI staining. PC-3 cells were placed in 6-well plates, and after 24 h of incubation, cells were treated with BEPT II-1 for 24 h and then harvested. After centrifugation, cell pellets were washed twice with cold phosphate-buffered saline. Cells were then incubated with 5 μL of annexin V-FITC and 10 μL of PI at room temperature for 15 min in the dark. After incubation, 400 μL of 1 × binding buffer was added to each tube. The cells were immediately analyzed by FACS Calibur flow cytometry (Becton Dickinson, New York, NY, USA).

## 4. Conclusions

Our studies showed that BEPT II-1 significantly inhibited the proliferation of PC-3 cells by inducing apoptosis. BEPT II-1 might be useful as a potential therapeutic agent against prostate cancer. Further examination of the mechanisms of the anticancer actions of BEPT II-1 is under progress.

## References

[1] Hong H., Zhang Y., Sun J., Cai W. (2009). Positron emission tomography imaging of prostate cancer. Amino Acids.

[2] Braun K., Ehemann V., Wiessler M., Pipkorn R., Didinger B., Mueller G., Waldeck W. (2009). High-resolution flow cytometry: A suitable tool for monitoring aneuploid prostate cancer cells after TMZ and TMZ-BioShuttle treatment. Int. J. Med. Sci..

[3] Peyromaure M., Valéri A., Rebillard X., Beuzeboc P., Soulié M., Salomon L. (2009). Characteristics of prostate cancer in men less than 50-year-old. Prog. Urol..

[4] Ramakrishna N.R., de Weese T.L., Chung L.W.K., Isaacs W.B., Simons J.W. (2001). Prostate Cancer: Biology, Genetics, and the New Therapeutics.

[5] Gilligan T., Kantoff P.W. (2002). Chemotherapy for prostate cancer. Urology.

[6] Hoskin D.W., Ramamoorthy A. (2008). Studies on anticancer activities of antimicrobial peptides. Biochim. Biophys. Acta.

[7] Ma J.Y., Guo X.M., Hu J.M., Wang X. (2011). New antimicrobial peptides purified directly from *Bullacta exarata*. Afr. J. Pharm. Pharmacol..

[8] Sivalokanathan S., Vijayababu M.R., Balasubramanian M.P. (2006). Effects of *Terminalia arjuna* bark extract on apoptosis of human hepatoma cell line HepG2. World J. Gastroenterol..

[9] Martin S.J., Green D.R. (1995). Apoptosis and cancer: The failure of controls on cell death and cell survival. Crit. Rev. Oncol. Hematol..

[10] Pan G., O’Rourke K., Chinnaiyan A.M., Gentz R., Ebner R., Ni J., Dixit V.M. (1997). The receptor for the cytotoxic ligand TRAIL. Science.

[11] Zapata J.M., Pawlowski K., Haas E., Ware C.F., Godzik A., Reed J.C. (2001). A diverse family of proteins containing tumor necrosis factor receptor-associated factor domains. J. Biol. Chem..

[12] Ashe P.C., Berry M.D. (2003). Apoptotic signaling cascades. Prog. Neuropsychopharmacol. Biol. Psychiatr..

